# Comparative study of growth responses and screening of inter-specific OTA production kinetics by *A. carbonarius* isolated from grapes

**DOI:** 10.3389/fmicb.2015.00502

**Published:** 2015-05-27

**Authors:** Iliada K. Lappa, Dimosthenis Kizis, Pantelis I. Natskoulis, Efstathios Z. Panagou

**Affiliations:** ^1^Laboratory of Microbiology and Biotechnology of Foods, Department of Food Science and Human Nutrition, Agricultural University of AthensAthens, Greece; ^2^Laboratory of Mycology, Department of Phytopathology, Benaki Phytopathological InstituteAthens, Greece

**Keywords:** *Aspergillus carbonarius*, OTA, growth assessment, modeling kinetics, predictive mycology

## Abstract

The aim of this work was to assess OchratoxinA (OTA) production of different *Aspergillus carbonarius* isolates, evaluate their growth profile through different growth measurements, and reveal any underlying correlation between them. Ten different isolates of *A. carbonarius* isolated from Greek vineyards located in different geographical regions were examined *in vitro* for their OTA production potential after an incubation period of up to 11 days. All fungal isolates grew on a synthetic grape juice medium (SGM) similar to grape composition at optimum conditions of temperature and water activity (25°C and 0.98 a_w_). Samples for OTA determination were removed at 3, 5, 7, 9, and 11 days of growth and analyzed by HPLC. Based on OTA measurements the isolates were characterized by diverse OTA production ranging from 50 to 2000 ppb at day 11. The different fungal growth responses (colony diameter, colony area, biomass, biomass dry weight, and colony density) have been measured and correlated with toxin production by means of principal components analysis (PCA), confirming satisfactory correlation and explained over 99% of data variability. Leudeking-Piret model was also used to study OTA production with time, revealing a mixed-growth associated trend and pointing a fail-safe model with slightly better prediction through colony area. This approach contributes to the assessment of correlation between mycotoxin production and different methods of fungal growth determination in relation to time.

## Introduction

Ochratoxin A (OTA) is a widely detected mycotoxin that was first described as a wine contaminant by Zimmerli and Dick (1995). Abarca et al. (1994) were the first that revealed the role of *Aspergillus* section *Nigri* and especially *Aspergillus carbonarius* in OTA production (Abarca et al., 2001). OTA is now known as a secondary metabolite produced by fungal species belonging to *Aspergillus* and *Penicillium* genera, that is related with nephrotoxic, hepatotoxic, genotoxic, teratogenic, and immunotoxic impact to humans and animals (IARC, 1993; Castegnaro et al., 1998). There is a great food safety concern regarding the presence of OTA in foods and thus the European Union has established maximum OTA levels of 2 μg Kg^−1^ for wine, grape juice, grape nectar, and grape must intended for direct human consumption and 10 μg Kg^−1^ for direct dried wine fruits (European Commission, 2006). Fungal isolates identification around the Mediterranean and other parts of the world have shown the occurrence of OTA-producing *Aspergillus* species in grapes. There is strong evidence of the significance of *A. carbonarius* in OTA production since there is high incidence of ochratoxin-producing isolates within *A. carbonarius* spp. (Stefanaki et al., 2003). Other studies showed that considerable climate differences related to geographical region influenced mould contamination and OTA (Cabañes et al., 2002; Visconti et al., 2008). Battilani et al. (2001) pointed that the major source of OTA in grapes is the skin of berries and considering that grape juices, musts and wines are produced by pressing berries, the diffusion ability of OTA becomes evident (Valero et al., 2006a), making thus OTA contamination a problem originating in the field. Black *Aspergilli* responsible for OTA are already present in vineyards (Tjamos et al., 2004) and the amount of toxin seems to be dependent on the latitude of the production (Battilani et al., 2006). The lower the latitude the more frequent the occurrence and the grater the concentration of the toxin (Rosari et al., 2000; Pietri et al., 2001; Chiotta et al., 2013).

Although, plenty of studies have examined the ecophysiology of *A. carbonarius* in different environmental conditions (Bellí et al., 2004; Tassou et al., 2009; Spadaro et al., 2010), few studies provide growth response data along with mycotoxin data for the same sampling time (Marin et al., 2005; Valero et al., 2006a). However, these studies focused on the examination of growth responses between different fungal species. During the last decade many publications on mycotoxin production over time have been reported on either synthetic media or food substrates, but comparisons between different fungal quantification methods have been rarely reported. However, Marin et al. (2005) correlated different growth responses among them but not with toxin production. Moreover, Garcia et al. (2013) tried to quantify the total amount of aflatoxins from maize and relate it to the amount of mold biomass. Hyphal extension rate is usually reported as radial growth rate (mm h^−1^) being probably the simplest and most direct measure of fungal growth. However, growth estimation for filamentous fungi from radial extension remains questionable due to differences in the height of mycelium and also its colony density (Taniwaki et al., 2006). In the quest of new methods for fungal quantification, the aim is to observe correlation among growth responses considering that biomass or diameter cannot be directly quantified in food systems (Marin et al., 2005). Garcia et al. (2013) used *Aspergillus flavus* as a model of mycotoxigenic fungus to relate aflatoxin to the amount of fungal biomass. Another attempt from Marin et al. (2008), demonstrated the correlation between colony diameter changes and toxin production in solid medium by means of model development. Fungal growth over time was investigated by Baranyi et al. (1993) who proposed a model that even though it had been developed for bacterial growth it was proved successful at fitting colony diameter increase (Gibson et al., 1994; Ghar et al., 2005). Mould growth was also empirically modeled with the modified Gompertz equation (Zwietering et al., 1990) selected for asymmetrical data (Ghar et al., 2005). Applying existing models to compare commonly employed parameters in growth assessment, as a fungal indicator will probably facilitate the establishment of secondary models. Moreover, correlation of OTA with different growth responses and fitting data to known models may be a step promoting predictive mycology.

The objectives of this study were to (i) compare different fungal growth responses, (ii) determine the effect of time on OTA production, (iii) correlate toxin with fungal growth under optimum temperature and a_w_ conditions, and (iv) screen inter-specific kinetics of toxin based on different growth approaches. To our knowledge this is the first attempt to describe multiple ochratoxin kinetics with the existence of growth associated (proportional to growth rate) and no growth associated (proportional to existing biomass/dry weight) production, and also correlate ochratoxin with a variety of growth responses concerning *A. carbonarius*.

## Materials and methods

### Fungal isolates and growth medium

Nine different wild isolates of *A. carbonarius* (coded as Ac27, Ac28, Ac29, Ac30, Ac31, Ac33, Ac34, Ac43, and Ac47) previously isolated from grapes collected from different geographical areas of Greece and a reference strain of *A. carbonarius* ITEM 5010 (Institute of Science of Food Production -ISPA, Bari, Italy) were used throughout this study. Isolates belonged to the fungal culture collection of the Laboratory of Food Microbiology and Biotechnology (LFMB) of the Agricultural University of Athens (stored in glycerol at −20°C). All isolates were tested for their potential for OTA production on Czapek yeast extract agar (CYA), after incubation at 25°C for 7 days as described by Kizis et al. (2014). OTA is classified as a possible human carcinogen within the 2B Group by IARC (IARC, 1993) and the related precautions were taken into account during laboratory work. The experiment was performed on Synthetic Grape juice Medium (SGM), a culture medium that simulates grape composition between véraison and ripeness (Delfini, 1982). Media were prepared by adding D(+) glucose, 70 g; D(–) fructose, 30 g; L(–) tartaric acid, 7 g; L(–) malic acid, 10 g; (NH_4_)H_2_PO_4_, 0.67 g; KH_2_PO_4_, 0.67 g; MgSO_4_ · 7H_2_O, 1.5 g; NaCl, 0.15 g; CaCl_2_, 0.15 g; CuCl_2_, 0.0015 g; FeSO_4_ · 7H_2_O, 0.021 g; ZnSO_4_ · 7H_2_O, 0.0075 g; (+) Catechin hydrate, 0.05 g; agar, 25 g, to 1000 ml distilled water. The *a_w_* of this basal medium was 0.98, measured by an AquaLab LITE (Degacon, USA) water activity meter at 25°C. The pH of the SGM was adjusted to 3.8 with KOH (2 M).

### Inoculation and incubation

Spore suspensions of each *A. carbonarius* isolate were prepared by collecting spores from 7-day old colonies grown on Malt Extract Agar at 25°C. Conidia were harvested from sub-cultures in an aqueous solution of 0.05% Tween 80 by scraping the surface of the mycelium. The final concentration of spores was assessed by a Neubauer counting chamber (Brand, Wertheim, Germany) and adjusted by appropriate dilutions to 10^6^ spores ml^−1^. Sterilized cellophane membranes were placed on the top of SGM agar plates in order to help biomass assessment. It has been shown that membrane allows fungus to obtain nutrients from the substrate and grow very similarly as with no cellophane layer (Ramos et al., 1999). Petri dishes were centrally single spotted with 10^3^ spores on the surface of the membrane. Incubation was performed at 25°C and *a*_w_ 0.98, which is optimum for *A. carbonarius* growth (Bellí et al., 2004; Garcia et al., 2010; Kapetanakou et al., 2011). Plates were sampled over time for the determination of biomass dry weight, colony radius, colony area, and OTA production, for a period of 11 days. All the assays were replicated in triplicate.

### Growth assessment

Colony diameter (mm), colony area (mm^2^), and biomass (mg dry weight) were measured at the same time in days 3, 5, 7, 9, and 11. Colony radius was observed on a daily basis and recorded at right angles by the aid of a ruler. Colony area was calculated by estimating the surface of the circle (π *R*^2^) formed by each fungal colony. The mycelium remained intact and collected from the cellophane membrane to monitor biomass. Fungal dry mass was determined by drying the mycelium at 105°C (Passanen et al., 1999), and measured after cooling at room temperature using desiccators (Taniwaki et al., 2006). Measurements were carried out periodically until weight was stabilized. Fungal biomass (mg) was recorded before drying as well. Finally, colony density was calculated by dividing mycelium dry weight by colony area (Marin et al., 2005; Garcia et al., 2013).

### Extraction and detection of ochratoxin A

The whole content of each plate was used for OTA extraction at 3, 5, 7, 9, and 11 days of incubation. Studies have indicated that OTA can be diffused throughout the culture medium, so taking into account the content of the Petri dish would ensure determination of the whole amount of OTA produced by the fungus (Valero et al., 2006b). Each sample was weighted and mixed with a 4-fold quantity of extraction solution (80% methanol: 20% water) using the Ultra Turrax (Heidolph Instruments, Schwabach, Germany) for 2 min at the highest speed (26 × 10^3^ rpm) (Kapetanakou et al., 2009). Extracts were filtered through a Whatman No2 filter paper, then through a 0.2 μm syringe-driven filter unit (Millex, Millipore Co., Bedford, Mass.) and stored at 4°C until HPLC analysis.

OTA was detected using an HPLC system equipped with a JASCO LC-Net II/ADC system controller, a JASCO AS-2055 Plus auto sampler, with a Model PU-980 Intelligent pump, a Model LG-980-02 ternary gradient unit pump and an FP-2020 Plus fluorescent detector (JASCO Inc., Easton, USA). The analysis was performed under isocratic conditions at a flow rate of 1 ml min ^−1^ of the mobile phase (water/acetonitrile/acetic acid; 49.5/49.5/1) through a Waters spherisorb C18 analytical column, 5 μm ODS2 (4.6 × 250 mm) (Resteck Co., Pinnacle II, Bellefonte, USA). Injection volume was 10 μl and run time for samples was 20 min with OTA detected at about 11 min. The detection limit of the analysis was 1 ppb (Bragulat et al., 2001).

### Statistical analysis

Analysis of variance (ANOVA) was performed allowing an overview of all the results and establishing correlations among the diverse growth parameters and OTA concentration of the different fungal isolates. The data set was analyzed by the statistical package JMP8 (SAS Institute Inc., Cary, NC, USA). Pearson's correlation matrix and descriptive statistics (means, standard deviations and coefficients of variance, CV%) were also computed by JMP8. Multivariate statistical analysis (Principal component analysis, PCA) was also employed to investigate any underlying relationship among the different variables through Statistica software ver. 8.0 (Statfoft, Tulsa, Oklahoma).

For ochratoxin modeling, the Leudeking-Piret mixed-growth associated model was used for product (OTA) formation as detailed previously by Garcia et al. (2013). In this work, fungal growth and OTA production were expressed by different equations depending on the fungal growth assessment parameter employed. Thus, when fungal growth was expressed as changes in diameter (X) vs. time, then growth and OTA production were fitted by the following equations:

$$\begin{array}{l} \text{If t}<\lambda ,\text{ X}=\text{0};\text{ P}=\text{0}\\ \text{If }\lambda <\text{t}<{\text{t}}_{\text{Xmax}},\text{ X}=\text{at}+\text{b};\text{ P}=\left(\text{a}\alpha +\text{b}\beta \right)\text{t}+\text{a}\beta \frac{{\text{t}}^{2}}{2} \end{array}$$

Where P is product concentration (g/L), α is the growth-associated coefficient for P production (gP/gX), β is the non-growth-associated coefficient for P production (gP/gX h), and t_Xmax_ is the time point where the linear model reaches its maximum value

For colony area (X), the respective equations are the following:

$$\begin{array}{l} \text{If t}<\lambda ,\text{ X}=\text{0};\text{P}=\text{0}\\ \text{If }\lambda <\text{t}<{\text{t}}_{\text{Xmax}},\text{ X}=\pi {\left(\text{at}+\text{b}\right)}^{2};\text{P}=\pi \beta {\text{a}}^{2}\frac{{\text{t}}^{3}}{3}\\ \;+\left(\pi \alpha {\text{a}}^{2}+\pi \beta \text{ba}\right){\text{t}}^{2}+\left(\text{2}\pi \alpha \text{ba}+\pi \beta {\text{b}}^{2}\right)\text{t} \end{array}$$

Finally, for biomass dry weight (X):

$$\begin{array}{l} \text{If t}<\lambda ,\text{ X}=0;\text{P}=0\\ \text{If }\lambda <\text{t}<{\text{t}}_{\text{Xmax}},\;X=ct^{2}+dt+e;\text{P}=\beta c\frac{t^{3}}{3}\\ \;+\left(\alpha c+\frac{\beta d}{2}\right)t^{2}+\left(\alpha d+\beta e\right)t \end{array}$$

Product formation (P) as well as fungal growth expressed as changes in diameter, colony area, and biomass dry weight were estimated by means of nonlinear regression based upon the Marquardt algorithm using Statgraphics® Centurion XV, version 15.1.02 (Statpoint, Inc., Maryland, USA).

## Results and discussion

### Growth responses

**Figure 1:**
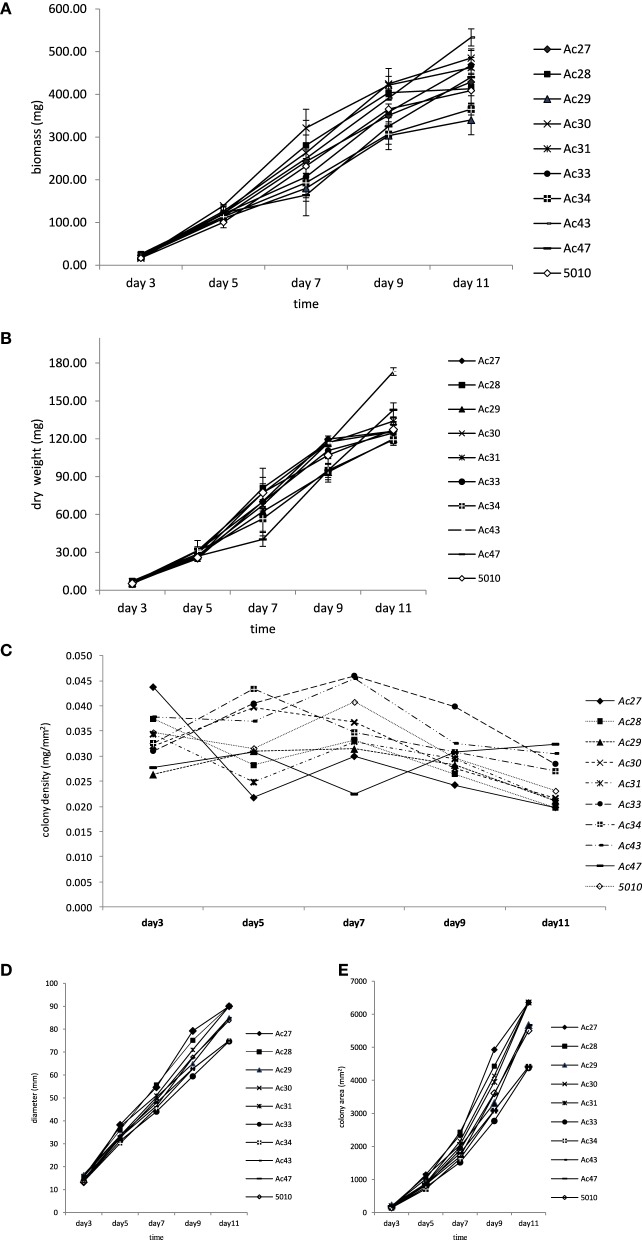
**Changes in growth responses of 10 isolates of** ***A. carbonarius*** **plotted against incubation time**. **(A)** Changes in biomass; **(B)** changes in dry weight; **(C)** changes in colony density; **(D)** changes in diameter; **(E)** changes in colony area; data points indicate mean values ± standard deviation of 3 replicates; for clarity of the figure no error bars were included when standard deviation was less than 5%.

Differences in fungal growth variables concerning colony diameter, biomass, mycelium dry weight, colony area, and colony density vs. time are presented in Figure 1. The influence of colony age on the data obtained by the five different methods used to measure fungal growth is also shown in this figure, where the actual values presented an increasing trend with incubation time (Figures 1A,B,D,E) with the exception of colony density (Figure 1C) where the opposite effect was evident. All five growth responses were found to be highly positively correlated among them, except colony density that was negatively correlated with the remaining growth parameters (Table 1). Similar significant positive correlations between dry weight and diameter of A. *carbonarius* were also confirmed by Marin et al. (2005) and of *Aspergillus flavus* by Garcia et al. (2013). Biomass dry weight correlation (*R*^2^ = 0.96–0.98) with diameter indicated that colony diameter in single cultures is an easy-to-use and acceptable choice for fungal growth estimation for research purposes even though it is difficult to be applied on food substrates. There are studies suggesting that colony diameter is not an acceptable indirect measure of fungal biomass since colonies seem to become denser with thicker morphologies in older areas (Wyatt et al., 1995; Taniwaki et al., 2006). Coefficients of variation (CV%) among growth responses were also estimated revealing higher dispersion for colony area and lower for mycelium diameter (Table 2). Moreover the values of the CV index indicated that day 3, corresponding to the early stage of fungal development, presented the highest variation compared to day 11, where all fungal isolates seemed to have similar growth responses (Table 2).

**Table 1:** **Correlation among growth responses (Pearson coefficients and corresponding** ***P*****-values in parentheses)**.

	**Diameter**	**Dry weight**	**Biomass**	**Colony area**	**Colony density**
Diameter	1	0.9528 (<0.0001)	0.9552 (<0.0001)	0.9763 (<0.0001)	−0.4784 (<0.0001)
Dry weight	0.9528 (<0.0001)	1	0.9858 (<0.0001)	0.9234 (<0.0001)	−0.2716 (0.0008)
Biomass	0.9552 (<0.0001)	0.9858 (<0.0001)	1	0.9201 (<0.0001)	−0.2975 (0.0002)
Colony area	0.9763 (<0.0001)	0.9234 (<0.0001)	0.9201 (<0.0001)	1	−0.5203 (<0.0001)
Colony density	−0.4784 (<0.0001)	−0.2716 (0.0008)	−0.2975 (0.0002)	−0.5203 (<0.0001)	1

**Table 2:** **Coefficients of variation (CV%) of growth responses along time**.

**Time (days)**	**Biomass CV%**	**Dry weight CV%**	**Diameter CV%**	**Colony area CV%**
3	17.55	18.28	12.44	24.76
5	9.40	14.05	10.85	22.07
7	21.55	17.11	10.06	20.11
9	11.93	9.52	10.58	21.08
11	13.02	12.93	9.56	17.98

### Ochratoxin data kinetics

**Figure 2:**
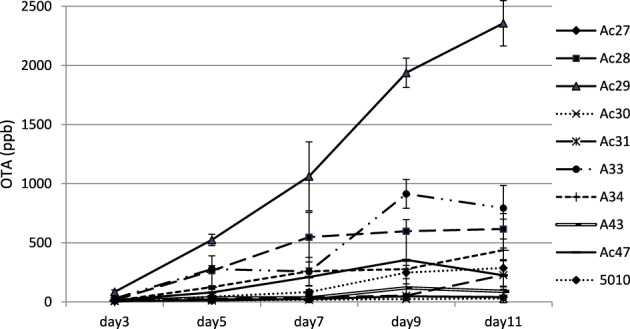
**Changes in OTA production over time for** ***A. carbonarius*** **isolates on SGM medium**.

Ochratoxin A was detected after 3 days of incubation and reached a maximum at 9–11 days (Figure 2). Regarding OTA production, fungal isolates could be discriminated into three broad classes containing low (<100 ng/g), medium (between 100 and 1000 ng/g) and high (>1000 ng/g) OTA producers at 11 days of incubation. Observation of OTA concentration in agar plates showed that the toxin increased with time reaching a plateau at the end of incubation period. However, for some strains maximum OTA levels were attained earlier followed by a decrease thereafter (Figure 2). This decrease was observed after the 9th day of mycelium growth could be attributed to toxin degradation by the fungus in an attempt to find an alternative carbon source to maintain its metabolic activity (Valero et al., 2006a). Analysis of variance for OTA production revealed that all single factors and their 2-way interactions were statistically significant at *p* < 0.001. The significance of the factor “isolate” was obviously biased by the differences in the OTA producing capacity of the different isolates studied. Analysis of variance for OTA between the different sampling times pointed an almost 2-fold higher effect of day 11 on OTA production than all the other days (data not shown). Moreover Coefficients of variation (CV%) revealed a wider dispersion of detected OTA production among isolates than among the 5 sampling days for each isolate (Table 3). This observation points the significance of inter-specificity of the *A. carbonarius* species. The analysis of variance pointed an almost 2-fold higher effect of day 11 on OTA production than all the other days. Few studies (Marín et al., 2006) have reported on the effect of incubation time on the amount of OTA produced. Contrary to long incubation periods employed by many authors, in the present study the focus was given on the early stage of fungal infection. As inferred by the analysis of variance, the significance of time on OTA production was extremely high, confirmed by the fact that day 11 seemed to have in most cases the highest effect. Moreover, observations of the CV index among isolates showed that decreasing the mean OTA producing ability resulted in increasing OTA dispersion, as illustrated by the CV values for isolates Ac27 and Ac30, with higher values observed at early days of production (Table 3). With regard to the isolates used in the current work, they were originated from the areas of Crete and Attica, corresponding both to a geographical localization of low altitude and hot and dry regions. As mentioned before, several reports point also the impact of region and climate (Visconti et al., 2008; Perrone et al., 2013) in *A. carbonarious* presence and OTA production in grapes. In addition to that, there are similar works indicating the importance between different grape cultivation practices (Tjamos et al., 2004; Bau et al., 2005). In the present work one isolate presented significantly higher toxin production, even from the early stage of growth, among all isolates. This particular isolate, Ac29, was originated from the same vineyard as isolate Ac27, which presented a 40-fold lower OTA production. So the variability in toxin potential between these specific isolates cannot be explained exclusively in terms of geographical location and cultivation practices and hence further investigation is necessary in terms of OTA related genetic factors. The focus of the present work was the *in vitro* investigation of the growth pattern of *A. carbonarius* in a growth medium resembling grape juice between véraison and ripeness, since this fungus has been isolated more frequently during this period in Greece (Tjamos et al., 2006; Meletis et al., 2007). Since grapes are considered one of the greatest sources of OTA contamination by these fungi, it is crucial to know their growth behavior and correlation with toxin production.

**Table 3:** **OTA dispersion and CV% values**.

**Isolates**	**Day 3**	**Day 5**	**Day 7**	**Day 9**	**Day 11**	**MO**
Ac30	106.01	111.81	132.62	5.40	32.07	77.58
Ac27	113.37	86.09	109.03	24.77	13.92	69.43
Ac43	74.54	1.80	90.8	65.42	49.54	56.42
Ac31	56.48	8.50	68.37	24.01	70.81	45.63
5010	94.44	24.71	27.22	38.47	24.73	41.91
Ac47	27.58	34.92	36.18	94.49	102.98	59.23
Ac34	78.42	7.20	46.32	12.02	70.81	42.95
Ac33	45.34	10.62	3.60	13.38	24.16	19.42
Ac28	31.81	47.81	38.24	1.54	13.57	26.59
Ac29	27.12	29.05	33.16	38.18	22.49	32.00

### Correlation between growth and OTA production

**Figure 3:**
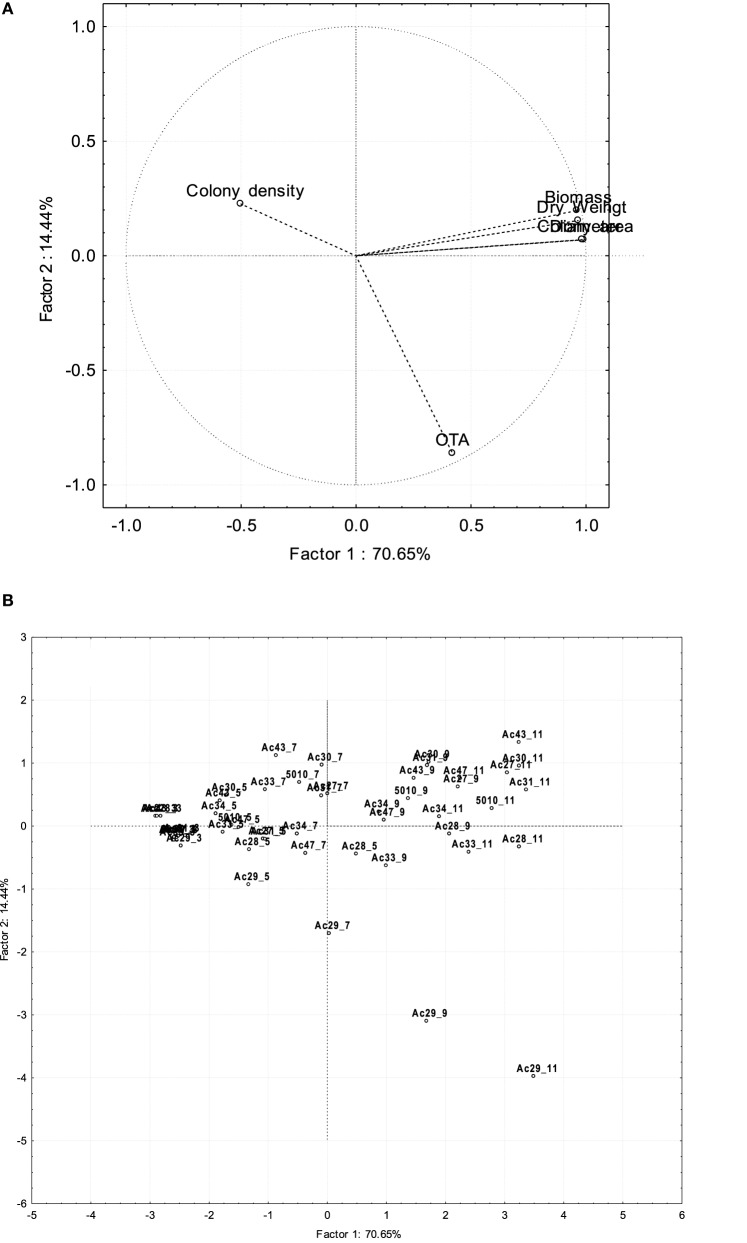
**Plot of loadings (A) and scores (B) resulting from PCA analysis for growth and OTA production of** ***A. carbonarius*** **isolates considering time of 5 incubation days**.

A statistically significant positive correlation (*p* < 0.05) was observed between most isolates' OTA production and growth responses on SGM, as derived by the Pearson correlation coefficients (Table 4), with the exception of colony density where a negative correlation was noticeable. OTA production was found to be correlated with each growth response parameter for 10 of the isolates examined. The higher OTA producers (isolates Ac28, Ac29, and Ac33) seemed to be better correlated with the measured growth parameters (*p* < 0.001) compared to the rest of the isolates. Principal component analysis (PCA) was performed taking as variables all 10 fungal isolates, the different growth measurements, OTA levels, and sampling times, confirming the correlation reported above (Figure 3). PC1 explained 70.6% of the variability in the dataset and it was positively correlated with all growth variables except colony density. PC2 explained the second larger variation in the dataset (14.4%) and it was associated with OTA production, including mainly the group of high OTA producer isolates. Finally, PC3 explained 13.91% of the variability and it was related to colony density. From the plot of scores (Figure 3), it can be inferred that Ac29 was highly correlated with OTA presenting an increasing trend with time. The first PC was related to time as there was a gradual transition of the growth assessment parameters from the left to the right quadrant of the plot, corresponding from low to high incubation times. Valero et al. (2006a) also showed correlation between colony radius and OTA production using also an isolate of *A. carbonarius*, however this research highlights the great impact of diverse fungal isolates (inter-specific variability) on OTA production.

**Table 4:** **Correlation among OTA production and growth responses (Pearson coefficients)**.

**Isolate**	**Diameter mm**	**Dry weight mg**	**Biomass mg**	**Area mm^2^**
Ac27	0.7370^*^	0.7847^*^	0.7409^*^	0.722^*^
Ac28	0.8725^**^	0.8923^**^	0.8949^**^	0.7994^*^
Ac29	0.8932^**^	0.9015^**^	0.8942^**^	0.8658^**^
Ac30	0.5913^*^	0.5771^*^	0.5870^*^	0.5782^*^
Ac31	0.7213^*^	0.6642^*^	0.6817^*^	0.7896^*^
Ac33	0.8776^**^	0.8932^**^	0.8930^**^	0.8572^**^
Ac34	0.6963^*^	0.7640^*^	0.7497^*^	0.6508^*^
Ac43	0.6312^*^	0.6295^*^	0.6577^*^	0.5812^*^
Ac47	0.3092	0.5221	0.4831	0.1815

Significance

** P < 0.001,

* P < 0.05.

### Modeling ochratoxin a data

**Figure 4:**
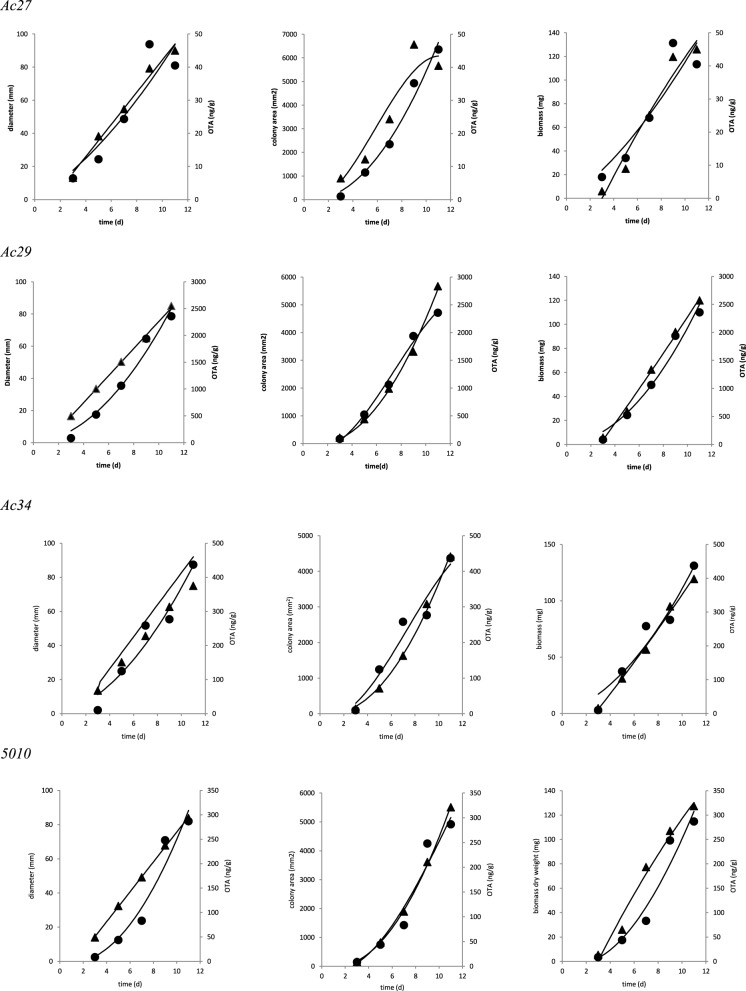
**Fitting of OTA (▲) concentration data to Leudeking-Piret models based on the different growth assessments (•)**.

**Figure 5:**
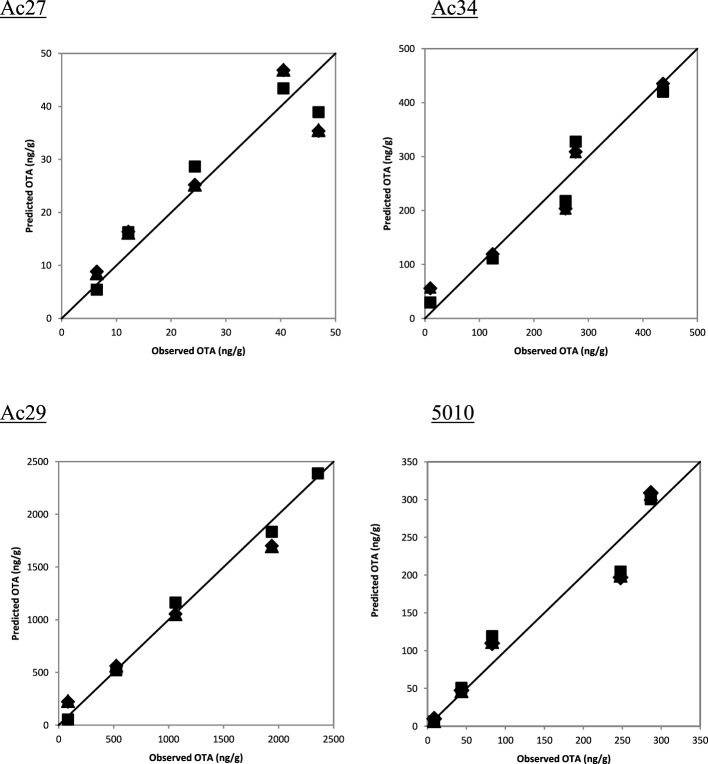
**Indicative diagrams of observed vs. predicted values of OTA through diameter (■), colony area (▲), biomass (♦)**.

Based on correlation results (Table 4), a selection in the initially employed fungal growth parameters was made and only three of them were finally taken into consideration, namely colony diameter, colony area, and biomass dry weight that presented the highest correlation with OTA. The selected growth parameters were further modeled using the Leudeking-Piret mixed-growth associated model to predict the amount of OTA produced in relation to incubation time. Table 5 shows the estimated model parameters and Figure 4 provides a graphical illustration of the fitting results for Ac27, Ac29, Ac34, and 5010. Generally, predicted OTA concentration through diameter, colony area, or biomass dry weight, led to a mixed-growth associated model, since no specific trend of toxin formation was predicted. Moreover, a low A_f_ mean value of 1.385 suggested small differences between predicted and observed data as presented in Figure 5. Also, B_f_mean values as 1.24 for colony diameter, 1.04 for colony area and 1.22 for biomass suggested a fail-safe model (Table 6). For isolates Ac31, Ac43, and Ac47 the Leudeking-Piret model could not predict OTA at day 3 for some of the growth parameters measured, so B_f_ and A_f_ values could not be estimated.

**Table 5:** **Parameters and standard errors concerning OTA estimated by the Leudeking-Piret model**.

	**Diameter**			**Area**			**Dry weight**		
	**α(ng/g mm)**	**β(ng/g mm d)**	***r*^2^**	**α(ng/g mm)**	**β(ng/g mm d)**	***r*^2^**	**α(ng/g mm)**	**β(ng/g mm d)**	***r*^2^**
Ac27	0.80 ± 0.30^*^	0.40 ± 0.31^*^	83.85	0.01 ± 0.004	0.003 ± 0.001^*^	91.10	0.17 ± 0.03	0.02 ± 0.02^*^	84.33
Ac28	6.01 ± 2.52^*^	0.14 ± 0.72^*^	82.11	0.38 ± 0.04	−0.08 ± 0.01	96.61	2.63 ± 0.53	0.31 ± 0.31^*^	83.10
Ac29	6.78 ± 4.11^*^	4.54 ± 1.14	97.30	0.94 ± 0.09	−0.15 ± 0.02	99.36	8.74 ± 1.46	2.52 ± 0.69	97.19
Ac30	0.24 ± 0.01	0.008 ± 0.003^*^	99.64	0.01 ± 0.004	0.003 ± 0.001^*^	82.28	0.09 ± 0.002	0.0003 ± 0.001	99.64
Ac31	−0.74 ± 0.85^*^	0.61 ± 0.24^*^	83.70	−0.02 ± 0.02^*^	0.01 ± 0.008^*^	93.00	0.16 ± 0.25^*^	0.16 ± 0.25^*^	83.70
Ac33	3.91 ± 5.18^*^	1.61 ± 1.43^*^	78.90	0.48 ± 0.22^*^	0.08 ± 0.07^*^	82.90	2.75 ± 1.00^*^	0.84 ± 0.57^*^	79.46
Ac34	2.17 ± 1.08^*^	0.67 ± 0.31^*^	94.14	0.19 ± 0.04^*^	0.01 ± 0.008^*^	95.16	1.86 ± 0.38	0.32 ± 0.17^*^	94.04
Ac43	0.62 ± 0.57^*^	0.11 ± 0.17^*^	70.45	0.06 ± 0.02^*^	−0.01 ± 0.009^*^	77.09	0.17 ± 1.11^*^	−0.46 ± 0.69^*^	52.14
Ac47	3.26 ± 2.64^*^	0.84 ± 0.66^*^	64.16	0.23 ± 0.07	0.04 ± 0.02^*^	80.92	12.20 ± 7.45^*^	−2.78 ± 2.15^*^	52.14
5010	0.27 ± 0.72^*^	0.70 ± 0.21	93.92	0.07 ± 0.03^*^	0.007 ± 0.03^*^	94.55	0.66 ± 0.17	0.38 ± 0.09	94.25

* not significant.

**Table 6:** **Accuracy and Bias factors arising from OTA data**.

	**Diameter**				**Colony area**				**Biomass dry weight**			
	**B_*f*_**	**A_*f*_**	**y**	***r*^2^**	**B_*f*_**	**A_*f*_**	**y**	***r*^2^**	**B_*f*_**	**A_*f*_**	**y**	***r*^2^**
Ac27	1.11	1.24	0.9573x	0.79	1.03	1.19	0.9765x	0.89	1.10	1.23	0.9585x	0.80
Ac28	1.56	1.80	0.955x	0.71	1.39	1.46	0.991x	0.95	1.54	1.77	0.958x	0.74
Ac29	1.21	1.28	0.9908x	0.97	0.92	1.13	0.9979x	0.99	1.22	1.29	0.9906x	0.96
Ac30	1.01	1.02	0.9994x	0.99	0.54	1.93	0.9703x	0.90	1.01	1.02	0.9995x	0.99
Ac33	1.32	1.63	0.9243x	0.73	1.18	1.40	0.9386x	0.78	1.29	1.60	0.9263x	0.74
Ac34	1.36	1.52	0.9824x	0.93	1.20	1.37	0.9855x	0.94	1.37	1.53	0.9822x	0.93
5010	1.08	1.18	0.975x	0.93	1.02	1.22	0.9776x	0.94	1.03	1.21	0.9763x	0.93

In our study OTA production in tandem with growth assessments, observations showed a decrease in concentration without any sign of decrease in mycelium growth. It must be stressed that as new plates were analyzed at each time period, an increase in the already recognized intrinsic variability in mycotoxin production was expected. Overall, modeling OTA concentration along time and taking inter-specificity into consideration, pointed a slightly better prediction through colony area.

## Conclusions

In conclusion, all growth responses studied were found to be correlated with each other. However, higher dispersion expressed as CV% was observed at the early stage of fungal development. Regarding OTA, dispersion was higher among isolates than between the sampling days, and also among isolates of lower ochratoxigenic potential. Multivariate statistical analysis showed that PCA explained more than 99% of the data-set variability in the 3 first PC with component 1 corresponding to the sampling time for all isolates.

In relation to Leudeking-Piret mixed-growth associated model, OTA production in the present study followed a rather mixed growth associated trend among the *A. carbonarius* isolates. Statistical indices of A_*f*_ and B_*f*_ for model performance suggested that the model is a safe approach for OTA prediction. The present work highlights that the dependence of the results from each method assayed lays not only at species level but also among species isolates. Due to the variability of *A. carbonarius* to diverse environmental conditions further research is needed to validate our results with additional experimental data. Understanding, and even more, predicting fungal growth, could become an important step in the evaluation and prediction of OTA production of *A. carbonarius*, since toxin formation was highly correlated with growth parameters. So if growth could be limited, OTA presence could also be limited.

A deeper understanding of isolates' diversity of this species may trigger a better intervention for toxin prevention in field, while the primary modeling approach could serve as a tool for generating secondary models, promoting predictions for a better toxin control.

## Author contributions

IL performed experiments, analyzed data and wrote the paper. DK and PN were involved in the experimental design of the work and interpretation of data. EP supervised the project, analyzed the data and revised the paper. All authors approved the final version of the manuscript to be submitted for publication and agreed to be accountable for all aspects of the work in ensuring that questions related to the accuracy and integrity of any part of the work are appropriately investigated and resolved.

### Conflict of interest statement

The authors declare that the research was conducted in the absence of any commercial or financial relationships that could be construed as a potential conflict of interest.
